# A Comparison of the Effectiveness of Combined Primer and Adhesive Systems in Orthodontic Bonding: An In Vitro Study

**DOI:** 10.3390/jcm14144892

**Published:** 2025-07-10

**Authors:** Filiz Uslu, Tugce Esra Gunes, Mehmet Akin, Hayri Akman

**Affiliations:** 1 Department of Orthodontics, Faculty of Dentistry, Alanya Alaaddin Keykubat University, 07400 Alanya, Antalya, Turkey; esra.gunes@alanya.edu.tr (T.E.G.); mehmet.akin@alanya.edu.tr (M.A.); 2 Department of Pediatric Dentistry, Faculty of Dentistry, Alanya Alaaddin Keykubat University, 07400 Alanya, Antalya, Turkey; hayri.akman@alanya.edu.tr

**Keywords:** adhesive remnant index, dental bonding, orthodontic adhesives, orthodontic brackets, shear bond strength

## Abstract

**Background**: Adhesive systems are important for achieving reliable and durable bracket bonding in orthodontic treatments. The purpose of this study is to compare the outcomes of a new one-step orthodontic bonding system that combines the primer and adhesive solutions. **Methods**: In this study, four groups were formed, each consisting of 20 first premolar teeth. Acid etching was applied to all teeth for 30 s, except in Group II. Group I included teeth where a single layer of primer was applied using the Transbond XT system before bracket bonding. Group II consisted of teeth bonded with brackets after using a self-etching primer with the Transbond XT system. Group III used GC Ortho Connect, a single-step adhesive that incorporates the primer within the adhesive itself. Group IV, a single layer of primer was applied before bonding with GC Ortho Connect. Shear bond strength (SBS) was assessed using one-way ANOVA and the Tukey-HSD test, while Adhesive Remnant Index (ARI) scores were analyzed using the Chi-square test at a significance level of *p* < 0.05. **Results**: SBS measurements were recorded as 13.28 ± 2.15 MPa for Group I, 11.06 ± 2.26 MPa for Group II, 10.37 ± 1.92 MPa for Group III, and 16.02 ± 2.17 MPa for Group IV. Statistical analysis using the Tukey test revealed significant differences in SBS values between Groups II and IV, as well as Groups III and IV (*p* < 0.05). All groups showed clinically acceptable bond strength, with Group IV demonstrating superior adhesion. Additionally, the chi-square test indicated a statistically significant variation in the ARI scores across all four groups (*p* < 0.05). **Conclusions**: The adhesive performance of the one-step GC Ortho Connect system is clinically comparable to Transbond XT. However, primer application is necessary to achieve optimal bond strength.

## 1. Introduction

Conventional dental composite bonding systems generally include three main components: an etching agent for enamel surface preparation, a primer, and an adhesive resin. Conventional total-etch systems are highly technique-sensitive and consist of three steps: etching, rinsing, and drying. Self-etching primers reduce this process to two steps (1. Etching + Primer, 2. Bonding) or can even simplify it into a single-step procedure (Etching + Primer + Bonding) [1,2]. Recent advancements in dental bonding materials have allowed for a reduction in procedural steps and overall application time, streamlining orthodontic bonding protocols. These advancements include the development of self-etching systems that combine etching and priming agents into a single solution [3,4], as well as systems that integrate etching agent, primer, and adhesive into a single, multifunctional product [5,6,7]. Buonocore was the first researcher to use high-concentration phosphoric acid (85%) for the purpose of acid etching dental surfaces. This method marked a major breakthrough in orthodontic bracket bonding, but it failed to create the honeycomb-like structure in the enamel prisms, which is essential for strong adhesion, resulting in inadequate bracket retention. Subsequent studies by Retief and colleagues demonstrated that using a 50% concentration of phosphoric acid resulted in a stronger bond. Today, phosphoric acid solutions with concentrations of 35–38% are widely used to enhance micromechanical bond strength and achieve surface modification of the enamel [8,9]. Studies indicate that the optimal duration for acid etching of the enamel surface is between 15 and 30 s, as shorter or longer application times may not provide sufficient bond strength [10].

The conventional orthodontic bonding procedure involves several essential steps, including cleaning and conditioning the enamel surface, applying a primer to the etched enamel, placing adhesive on the bracket base, and accurately positioning the bracket. From a clinical standpoint, longer bracket placement times increase the likelihood of bond failure due to inadequate moisture control [11]. To overcome this, orthodontic adhesives integrated with primers simplify the process by minimizing technical sensitivity during application. Additionally, the use of self-etch primers (SEPs), which both etch and primer the enamel in one step, makes the procedure easier [12,13].

Self-etching and self-adhesive bonding systems streamline the bonding procedure by minimizing the required steps, but this convenience is often accompanied by reduced bond strength, which can present a limitation in certain clinical situations. The durability of orthodontic devices against chewing forces is determined by shear bond strength, which is an important criterion in the development of adhesive materials. Some new bonding systems have been developed that combine the primer solution with the adhesive. The benefit of these systems is that they simplify the conventional bonding process by removing the second step, which involves primer application after the enamel is etched [14].

Shear Bond Strength (SBS) refers to the bonding strength between the bracket and the enamel surface, while Adhesive Remnant Index (ARI) indicates the amount of adhesive remaining on the enamel after bracket removal. Increasing SBS enhances bracket stability and reduces the risk of failure during treatment, while optimizing ARI helps protect the enamel and shortens the cleanup time. Improving both parameters together leads to more predictable outcomes and a more efficient treatment process.

Recent advancements in orthodontic adhesive technology have enabled the development of one-step systems—such as Biofix (Biodinamica Dental Products, Vinhos, Portugal), Heliosit Orthodontic (Ivoclar Vivadent, Schaan, Liechtenstein), and GC Ortho Connect (GC Orthodontics, Breckerfeld, Germany)—that require only the etching phase and eliminate the need for a separate primer application. This bonding paste contributes to stable bond formation through the inclusion of a phosphoric ester monomer and does not require the use of a separate primer. Additionally, its optimal fluorescence property allows for the detection of adhesive remnants on the tooth surface after bracket debonding using a light-curing device [15]. Among these systems, GC Ortho Connect was selected for this study due to its more extensive representation in the literature, both independently and in comparison with the Transbond XT system, thereby allowing for more reliable and meaningful comparative analysis of the obtained data [16,17,18]. However, current evidence on the clinical and laboratory performance of such systems, particularly with or without the use of an additional primer, remains limited. This study aims to address this gap by evaluating the effects of external primer application on the shear bond strength and adhesive remnant profile of the GC Ortho Connect system.

The null hypothesis of this study is as follows: there is no statistically significant difference in shear bond strength and adhesive remnant index between the primer-coated and uncoated forms of the GC Ortho Connect system and the conventional multi-step adhesive system in terms of bonding orthodontic brackets to the enamel surface.

## 2. Materials and Methods

This research was conducted with the approval of the Ethics Committee of the Faculty of Dentistry at Alanya Alaaddin Keykubat University (Approval Code: 4/8; Approval Date: 12 February 2025). Prior to conducting the study, a power analysis was carried out using G∗Power (version 3.0.10; Franz Faul University, Kiel, Germany) to determine the necessary sample size. Based on a 1:1 ratio between groups, a total sample size of 80 teeth was found to provide more than 85% power (true power = 0.8777) to detect significant differences with an effect size of 0.35 at a significance level of *p* < 0.05.

This study utilized a total of eighty non-carious and unrestored premolar teeth extracted for orthodontic purposes. The selection criteria for the teeth included the preservation of intact buccal enamel surfaces, absence of caries, and lack of previous restorations. The randomly selected teeth were divided into four experimental groups, each consisting of 20 teeth. The extracted teeth were stored in 0.1% thymol solution at room temperature until the experiments were conducted in order to prevent bacterial contamination and preserve the structural integrity of the dental tissues [19]. To ensure that only the crown portion remained exposed and that the labial surfaces were properly aligned with the applied force, the teeth were vertically embedded in self-curing acrylic. Before the experiments, the tooth surfaces were cleaned with a pumice and water mixture for 10 s and then thoroughly rinsed with water.

In this study, a total of eighty stainless steel premolar standard edgewise brackets (790–010, Dentaurum, Pforzheim, Germany) with an average base surface area of 10 mm^2^ were used. All brackets were carefully positioned by a single operator in the most appropriate location, perpendicular to the enamel surface, in accordance with the manufacturer’s instructions. Excess adhesive was meticulously removed in all groups. To ensure that the applied force was directed uniformly across all samples, the teeth were positioned upright, aiming for proper alignment between the bracket base and the loading plate. This setup enabled standardized axial force application across all specimens.

Two different orthodontic adhesive systems were utilized in this study: Transbond XT (3M Unitek, Monrovia, CA, USA) and GC Ortho Connect (GC Orthodontics, Breckerfeld, Germany). Transbond XT is a light-cured, resin-based orthodontic adhesive. It contains Bis-GMA and TEGDMA monomers, along with silane-treated quartz fillers. Due to its high bond strength and ease of application, it is widely preferred in clinical orthodontics. GC Ortho Connect is a resin-based orthodontic adhesive system with a single-step application feature. It contains phosphoric ester monomers (MDPs) and bis-methacrylate monomers. This composition supports chemical bonding with the enamel while providing high bond strength.

The groups in the study were formed as follows:

Group I: The tooth surfaces in this group were etched for 30 s using 37% orthophosphoric acid (i-GEL, Aviacijos, Siauliai, Lithuania). Following the etching process, a single layer of XT Primer (3M Unitek, Monrovia, CA, USA) was applied to each tooth using a dental microbrush. The brackets were positioned on the tooth surfaces using Transbond XT composite resin and light-cured for 20 s with a light-emitting diode (LED) curing unit in accordance with the manufacturer’s instructions.

Group II: In this group, a self-etching primer was applied to 20 teeth prior to bracket bonding with the Transbond XT system. The adhesive was then light-cured, following the same polymerization protocol as used in Group I.

Group III: In this group, the teeth were pretreated by etching with 37% orthophosphoric acid (i-GEL, Aviacijos, Siauliai, Lithuania) for 30 s to prepare the enamel surface. After etching and drying, a single-step adhesive containing a built-in primer, GC Ortho Connect (GC Orthodontics, Breckerfeld, Germany), was applied to bond the brackets.

Group IV: In this group, the 20 teeth were etched with 37% orthophosphoric acid prior to bonding the brackets with GC Ortho Connect adhesive (GC Orthodontics, Breckerfeld, Germany). Following the etching process, a single layer of XT Primer (3M Unitek, Monrovia, CA, USA) was applied to the tooth surfaces. The use of XT Primer in Group IV represents an off-label application of the GC Ortho Connect system, performed to evaluate whether primer addition enhances bond strength. Similarly to Group I, the brackets were then light-cured after positioning.

### 2.1. Debonding Procedure

After bracket bonding, the specimens were stored in distilled water at 37 °C for 24 h and then subjected to 10,000 thermal cycles between 5 °C and 55 °C (±2 °C), with a dwell time of 20 s for each cycle, to simulate intraoral conditions. For shear bond strength testing, a steel knife-edged chisel was positioned 0.2 mm away from the bonded interface in a universal testing machine (TSTM 02500; Elista Elektronik Informatik Sistem Tasarım Ltd. Şti., Istanbul, Turkey), which was calibrated according to the manufacturer’s guidelines prior to use. Load was applied at a crosshead speed of 0.5 mm/min. The SBS value, expressed in megapascals (MPa), was automatically calculated by dividing the failure load (N) by the bonded surface area (mm^2^).

### 2.2. Remnant Adhesive

**Figure 1:**
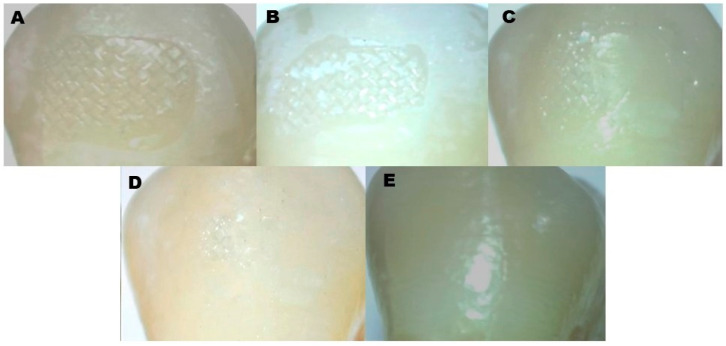
(**A**): ARI score 1; (**B**): ARI score 2; (**C**): ARI score 3; (**D**): ARI: score 4; (**E**): ARI score 5.

All samples and brackets were examined under 10x magnification after debonding to determine the adhesive remnant index scores. The adhesive remaining on the tooth was evaluated using the ARI [20,21] scoring system and categorized according to the ARI criteria. The ARI scale ranges from 1 to 5 (Figure 1).

1. An impression of the bracket base and all of the composite was still present on the tooth.

2. Over 90% of the composite material was still present on the tooth.

3. Less than 90% of the composite was still present on the tooth, but more than 10% was.

4. The composite’s retention on the tooth was less than 10%.

5. There was no composite remaining on the enamel.

The locations of bond failure between the enamel, adhesive, and bracket base were identified in further detail using the ARI scores.

### 2.3. Statistical Analysis

Statistical calculations were performed with SPSS (version 21.0; SPSS, Chicago, Il, USA). For each of the four groups, descriptive statistics such as the mean, standard deviation, minimum, and maximum values were calculated. It was examined whether the data followed a normal distribution using the Kolmogorov–Smirnov test, and the results indicated that the data were normally distributed and that there was homogeneity of variance among the groups. Therefore, statistical evaluation was performed using parametric tests. One-way analysis of variance (ANOVA) and Tukey’s Honestly Significant Difference (HSD) post hoc test were used to compare the SBS values among the groups. The inter-examiner reliability of ARI was evaluated with Cohen’s Kappa test (0.84). The ARI between the groups was compared using the chi-square test. Possible associations between SBS and ARI were assessed using Pearson correlation. *p* < 0.05 was used as the statistical significance criteria for this study.

## 3. Results

### 3.1. Shear Bond Strength

The SBS values of the four groups are presented in Table 1. The mean SBS values were 13.28 ± 2.15 MPa for Group I, 11.06 ± 2.26 MPa for Group II, 10.37 ± 1.92 MPa for Group III, and 16.02 ± 2.17 MPa for Group IV. According to the Tukey Test, the only significant difference was found between Group II and Group IV and Group III and Group IV (*p* < 0.05). Additionally, the effect size between Group I (Transbond XT with primer) and Group III (GC Ortho Connect without primer) was calculated using Cohen’s d and found to be 1.43, indicating a large effect size.

### 3.2. Adhesive Remnant Index

According to the chi-square test results (χ^2^ = 29.867), a significant difference was found among the four groups in terms of ARI (*p* < 0.05). Table 2 shows the amount of adhesive remnant remaining on the bracket base and tooth after bracket debonding and the failure of the adhesive. In Groups II and III, composite remnants were predominantly found on the bracket base. The prevalence of ARI scores of 4 and 5 in these groups indicates that failures primarily occurred at the adhesive-enamel interface. In Group IV, composite remnants were mostly observed on the enamel surface. The occurrence of ARI scores of 1 and 2 in Groups IV and I suggests that failures primarily took place at the adhesive-bracket base interface (Table 2). Clinically, higher adhesive remnants on enamel (lower ARI scores) may increase cleanup time but reduce enamel damage risk, whereas lower adhesive remnants (higher ARI scores) facilitate faster cleanup but may pose higher enamel risk. Thus, the observed differences have practical implications for balancing enamel protection and clinical efficiency.

## 4. Discussion

In this study, the null hypothesis stating that the application of a primer layer does not affect shear bond strength and adhesive remnant index was rejected. The findings provide insight into the adhesive performance of this newer system, especially regarding shear bond strength and adhesive remnant characteristics, which are crucial for maintaining bracket retention without causing damage to the enamel during debonding.

Orthodontic brackets bonded to enamel surfaces have a clinically limited lifespan. To ensure secure and long-term retention, the bond strength of the adhesives used should be sufficient to maintain stable bracket attachment while also allowing for safe debonding without causing damage to the enamel. Additionally, any residual adhesive remaining after debonding should be easily and efficiently removable [22]. In this study, we aimed to evaluate the bond strength of a one-step orthodontic adhesive system against conventional total-etch and self-etch systems, taking into account the cost-effectiveness and prevalent application of Transbond orthodontic adhesive.

A previous study reported that etching the enamel surface with 37% phosphoric acid for 30 s resulted in higher bond strength compared to a 15 s application. However, it has also been noted that etching durations longer than 30 s may reduce bond strength and potentially damage the enamel surface. Therefore, in the present study, an etching time of 30 s was selected, as it is considered the most appropriate duration in the literature [23].

Clinical studies have reported orthodontic bracket failure rates ranging between 4% and 6%. In this study, the aim was to enhance the clinical effectiveness and reliability of orthodontic treatment by increasing the success rate of bracket bonding [24,25].

Not using primer during the bonding stage is time-efficient because one step is eliminated. This can be critical during bracket placement, as the procedure takes longer, and the risk of moisture contamination disrupting the bond increases. Additionally, eliminating one step provides cost savings and reduces the time the orthodontist spends in the chair [26,27]. In this study, the bond strength of the one-step orthodontic adhesive system with and without primer was measured, and the difference between the two applications was found to be statistically significant. Findings indicating that the application of a primer layer between the tooth surface and the adhesive can enhance SBS suggest that this approach may offer a clinically advantageous method for achieving stronger adhesion. These results are consistent with the findings of Shapinko et al., supporting the positive impact of primer application on bonding performance [14].

The mean SBS values were found to be 13.28 ± 2.15 MPa for Group I, 11.06 ± 2.26 MPa for Group II, 10.37 ± 1.92 MPa for Group III, and 16.02 ± 2.17 MPa for Group IV. The results showed that Group IV, which utilized the GC Ortho Connect system with an additional layer of primer, demonstrated the highest mean SBS (16.02 ± 2.17 MPa), significantly higher than Groups II and III. This finding suggests that the application of an extra primer layer with a one-step adhesive system enhances bond strength, possibly due to increased penetration and mechanical interlocking at the enamel–adhesive interface. However, no significant difference was observed between Groups I and IV, indicating that while GC Ortho Connect is effective, its performance is comparable to the standard Transbond XT system with a primer layer. The relatively lower bond strength observed in Group III (GC Ortho Connect without additional primer) may be attributed to insufficient infiltration of the self-etching primer monomers within the adhesive into the enamel prisms due to the absence of an external primer layer. In other studies, where orthodontic adhesives were tested, lower [14,28] or higher [29] values were found. The obtained SBS values fall within the range of 5.9–7.8 MPa, which is the clinically acceptable bond strength range for orthodontic use reported in the literature. It has been found that high shear bond strength can lead to fractures or cracks in the enamel during debonding. Therefore, some researchers have suggested that the shear bond strength should not exceed 20 MPa [30,31]. This suggests that the GC Ortho Connect system provides secure bonding of brackets with or without an additional primer layer while offering clinical efficiency and ease of bracket removal [30].

High SBS ensures firm adhesion of the bracket to the enamel, thereby reducing the risk of bracket failure during treatment. This has been observed with the GC Ortho Connect system when used with primer. However, such high bond strength may increase the risk of enamel damage during debonding. Therefore, it is recommended that this balance be carefully considered. However, in this study, microscopic examination revealed no damage to the enamel surface following debonding.

Similarly to this current study, a few reports in the literature have shown that the GC Ortho Connect system provides clinically acceptable bond strength and does not demonstrate a significant difference when compared to multi-step systems such as Transbond XT. For example, in vitro studies [16,32,33] have indicated that GC Ortho Connect demonstrates comparable SBS values to Transbond XT. Such findings suggest that single-step systems may offer similar performance with reduced application time. However, it was noted that the application of an additional primer significantly enhanced bond strength [32]. Moreover, based on the available studies [16,32,33], it was concluded that, just like in this study, the GC Ortho Connect system provided clinically acceptable bond strength. Studies comparing GC Ortho Connect with other one-step orthodontic adhesive systems have reported no statistically significant difference in clinical failure rates [16].

The Chi-square test shows a significant difference between the four groups (*p* < 0.05). This indicates that there are differences between the groups in the distribution of adhesive remnants on the enamel or at the base of the bracket. In Groups II and III, mostly composite remnants were seen at the base of the bracket. ARI scores of 4 and 5 were more common in these groups, indicating that failures occurred at the adhesive–enamel interface. The adhesive remaining on the enamel can make the tooth difficult to clean. Observations on Groups IV and I reveal that mostly composite remnants are on the enamel surface. ARI scores of 1 and 2 are more common in these groups, indicating that failures occur at the adhesive–bracket base interface. It is hypothesized that the phosphoric ester monomers in the formulation of GC Ortho Connect may chemically interact with the external primer to form a dual-layer bonding interface. This interaction could enhance bond stability while also increasing the tendency for adhesive remnants to remain on the surface during debonding. This may explain the lower ARI scores observed in the groups where primer was used.

In this current study, similar to the study by Kazlauskaitė et al. [34], Transbond XT left more adhesive remnant on the tooth surface than GC Ortho Connect. This may be because GC Ortho Connect is a one-step system that includes a primer. As seen in this study, the addition of primer increases bond strength; however, it also tends to leave more adhesive remnant on the tooth surface.

As the ARI value increases, the amount of composite remnants on the tooth surface decreases. This allows for the earlier removal of remaining composite, thereby enhancing time efficiency [16]. Bishara emphasized that bond failure can be tolerated in the adhesive layer or at the bracket-adhesive interface, but it is unacceptable at the enamel–adhesive interface due to the risk of enamel surface damage during the debonding process [35]. The amount of remnant left by the adhesive material on the enamel surface can prevent traumatic microfractures, but in this case, it takes longer to clean, restore, and polish the tooth surface [36].

Improving SBS contributes to the long-term stability of orthodontic brackets by minimizing the risk of bracket failure during treatment, thereby reducing the need for unexpected clinical interventions. This enhances treatment efficiency and directly supports patient satisfaction. On the other hand, optimizing the ARI helps minimize the amount of residual adhesive on the enamel surface after debonding. As a result, the cleaning process becomes faster and easier, enamel damage is reduced, and chair time is shortened during bracket removal. Together, enhancing SBS and optimizing ARI leads to more predictable clinical outcomes, better preservation of enamel integrity, and a more efficient clinical workflow.

Vando Ribeiro-Neto et al. [37] demonstrated in their study that SBS decreased after thermocycling. This research indicates that thermal stress may have a negative impact on the bond stability of adhesive systems and that bond strength assessed under laboratory settings may reduce over time in a clinical environment.

### Limitations of the Study

This study has several limitations: Because it was conducted in a laboratory setting, it does not fully reflect true intraoral parameters such as saliva, temperature, and masticatory forces, limiting its capacity to provide direct insight into long-term clinical performance. The study only evaluated two adhesive solutions (Transbond XT and GC Ortho Connect), and the sample size was small, limiting the findings’ generalizability. All brackets were placed by a single skilled operator, but the lack of a mechanical positioning device or objective repeatability measurement limits sample standardization. Furthermore, clinical variables such as patient comfort, bracket removal time, and enamel damage were not evaluated.

Future randomized controlled clinical trials including different bracket types and operators with varying clinical experience are needed. Furthermore, microscopic analyses (such as SEM/EDS) investigating the bonding mechanisms may provide critical insights into the efficacy of the GC Ortho Connect system when a primer is employed.

## 5. Conclusions

This study shows that the GC Ortho Connect adhesive technology is a good option for successfully and safely attaching orthodontic brackets to enamel surfaces. The bond strength is slightly increased by applying an additional primer layer. The GC Ortho Connect system has the potential to simplify the bonding process and, in clinical settings, offers bond strength comparable to conventional primer and adhesive combinations. It could be beneficial to apply an additional primer layer, especially when a stronger bond is needed. Nevertheless, it is critical to maintain a balance between achieving suitable bond strength and providing safe bracket debonding without damaging the enamel surface. However, further study is required to better understand the long-term clinical performance and the long term of this adhesive solution.

## Figures and Tables

**Table 1:** Descriptive statistics and the results of ANOVA and Tukey’s comparing SBS.

Groups	n	Mean	SD	Min–Max	Anova	Tukey	Group II	Group III	Group IV
Primer + Transbond XT composite (Group I)	20	13.28	2.15	10.44–14.28	*p* = 0.002	AB	*p* > 0.05	*p* > 0.05	*p* > 0.05
SEP + Transbond XT composite (Group II)	20	11.06	2.26	9.16–14.50		A		*p* > 0.05	*p* = 0.034
GC Ortho Connect adhesive (Group III)	20	10.37	1.92	9.23–14.68	*f* = 23.51	A			*p* = 0.021
Primer + GC Ortho Connect adhesive (Group IV)	20	16.02	2.17	13.01–19.66		B			

**Table 2:** Frequency of distributions and comparison of ARI scores.

Groups	n	1	2	3	4	5
Primer + Transbond XT composite	20	8	8	4	0	0
SEP + Transbond XT composite	20	0	3	3	4	10
GC Ortho Connect adhesive	20	0	2	4	3	11
Primer + GC Ortho Connect adhesive	20	9	9	2	0	0

## Data Availability

The data presented in this study are available on request from the corresponding author due to privacy reasons.

## Abbreviations

The following abbreviations are used in this manuscript:

Adhesive Remnant Index	ARI
Megapascals	MPa
Shear bond strength	SBS
Self-etch primers	SEPs
